# Fathers' experiences of supporting breastfeeding: challenges for breastfeeding promotion and education

**DOI:** 10.1111/mcn.12129

**Published:** 2014-04-10

**Authors:** Amy Brown, Ruth Davies

**Affiliations:** ^1^ Department of Public Health and Policy Studies Swansea University Swansea UK; ^2^ Department of Nursing Swansea University Swansea UK

**Keywords:** fathers, breastfeeding, qualitative study, involving, education, health promotion

## Abstract

Increasing breastfeeding rates is a strategic priority in the UK and understanding the factors that promote and encourage breastfeeding is critical to achieving this. It is established that women who have strong social support from their partner are more likely to initiate and continue breastfeeding. However, little research has explored the fathers' role in breastfeeding support and more importantly, the information and guidance he may need. In the current study, 117 men whose partner had given birth in the previous 2 years and initiated breastfeeding at birth completed an open‐ended questionnaire exploring their experiences of breastfeeding, the information and support they received and their ideas for future breastfeeding education and promotion aimed at fathers and families. Overall, the findings showed that fathers were encouraging of breastfeeding and wanted to be able to support their partner. However, they often felt left out of the breastfeeding relationships and helpless to support their partner at this time. Many reported being excluded from antenatal breastfeeding education or being considered unimportant in post‐natal support. Men wanted more information about breastfeeding to be directed towards them alongside ideas about how they could practically support their partner. The importance of support mechanisms for themselves during this time was also raised. The results highlight the need for health professionals to direct support and information towards fathers as well as the mother–infant dyad and to recognise their importance in promoting and enabling breastfeeding.

## Background

An integral debate within the early parenting literature is how an infant is fed during the first months of life. Breastfeeding has numerous benefits for both infant and maternal health with formula fed infants at increased risk for gastrointestinal, respiratory and ear infections, obesity and atopic disease (Kramer & Kakuma 2002; Horta *et al*. 2007; Ip *et al*. 2007). Breastfeeding rates in the UK are however low (McAndrew *et al*. 2012), with considerable research highlighting the maternal difficulties, anxieties and attitudes that prevent breastfeeding success (Li *et al*. 2008; Thulier & Mercer 2009; Brown *et al*. 2011b). Specifically, the need for mothers to receive relevant and effective information and education has been highlighted. Mothers who feel informed, confident and knowledgeable about breastfeeding are more likely to both initiate and continue to breastfeed (Avery *et al*. 2009; Brown *et al*. 2011c; McQueen *et al*. 2011).

Alongside this, the importance of support has been highlighted as crucial to raising breastfeeding rates. High‐quality professional support and guidance is often referred to by mothers as being an important element of breastfeeding success (Brown *et al*. 2011c; Hauck *et al*. 2011; Schmied *et al*. 2011) but the role a woman's partner plays in both her decision to initiate and ability to continue breastfeeding is also critical. Mothers who have a partner who is supportive and encouraging are more likely to plan to breastfeed (Persad & Mensinger 2008), breastfeed on discharge from hospital (Scott *et al*. 2001) and to breastfeed for a longer duration (Gage & Kirk 2002; Garfield & Isacco 2006; Brown & Lee 2011). Specifically, higher levels of paternal support and encouragement are associated with greater maternal confidence to breastfeed (Hauck 2004; Swanson & Power 2005; Hauck *et al*. 2007) and mothers whose partner is supportive report feeling more capable and competent in breastfeeding decisions and challenges (Mannion *et al*. 2013).

However, given the importance of this relationship, there has been little consideration of fathers' needs and difficulties encountered in supporting their partner to breastfeed. Research suggests that fathers are interested in breastfeeding and want to be involved with and support the mother through this time (Sherriff *et al*. 2009, 2013; Sherriff & Hall 2011). However, fathers often feel unprepared for the challenges breastfeeding can bring (Sherriff *et al*. 2009), unsure how to help (de Montigny & Lacharite 2004) or feel left out, helpless and excluded from the experience (Pontes *et al*. 2009; Mitchell‐Box & Braun 2012). Fears of appearing predatory, or even simply having little experience of seeing breastfeeding and how it works arise (Henderson *et al*. 2011). Overall, fathers report wanting more information and support so that they can be proactively and effectively involved at this time (Sherriff *et al*. 2009, 2013; Tohotoa *et al*. 2009).

Emphasis has been placed on educating and promoting the importance of breastfeeding to new mothers, potentially finding new and innovative ways to encourage greater initiation and duration (Stewart‐Knox *et al*. 2003; Dykes & Flacking 2010). However, educating and supporting those who support the mother is also an important step (Powell & Baic 2011). Programmes so far aimed at involving fathers in breastfeeding have enjoyed mixed success. Although one randomised controlled trial in Australia found an increase in breastfeeding rates when fathers attended an antenatal and post‐natal support class (Maycock *et al*. 2013), other studies have not shown an impact of antenatal breastfeeding education for fathers upon breastfeeding rates (Susin & Giugliani 2008; Lovera *et al*. 2010). However, given the desire of fathers to be involved in the breastfeeding relationship, understanding fathers' experiences and needs at this time is important.

The aim of the current research was thus to explore fathers' experiences of supporting their partner during breastfeeding and to examine their attitudes towards the education, information and support they received. Specifically, ideas for how they would like to see future breastfeeding support for new fathers was investigated.

### Key messages

- Fathers want to be involved and support their partners in breastfeeding but many feel left out and helpless.
- Father want specific and accessible information about the benefits of breastfeeding, strategies to encourage and support their partner alongside support for themselves during this time.
- Health professionals must involve, include and support fathers, recognising their importance in the breastfeeding relationship.

## Methodology

### Design

A cross‐sectional, self report questionnaire.

### Participants

Men whose partner had given birth in the last 2 years and had initiated breastfeeding at birth were recruited via local mother and baby groups, online parenting forums based in the UK and social media methods such as online blogs, Facebook and Twitter. As fathers typically do not attend parenting groups and other stereotypically female venues that mothers often attend, mothers were encouraged to pass on details of the study to their partners. A 2‐year limit was chosen based on World Health Organization recommendations to breastfeed for the first 2 years post‐partum and beyond (WHO 2003) Participants provided details regarding occupation, which were coded according to the National Statistics Socio‐Economic Classification self‐coded method (NS‐SEC 2005). Fathers were all resident in the UK and provided UK postcode to confirm this.

All participants gave informed consent prior to inclusion in the study. Ethics approval was granted by a Department of Psychology Research Ethics Committee. All aspects of this study have been performed in accordance with the ethical standards set out in the 1964 Declaration of Helsinki.

### Questionnaire

Participants completed a questionnaire consisting of open‐ended questions examining their attitudes towards breastfeeding, beliefs regarding their experience in supporting their partner to breastfeed and attitudes towards breastfeeding promotion, education and support in the UK (Table 1). Questions examined both participant experiences and how fathers believed breastfeeding should be promoted. Questions were developed to specifically ask fathers to describe their experiences and give details of information they had received. Men also estimated their partners' breastfeeding duration and gave details of infant age and their demographic background (age, education, occupation, marital status).

**Table 1:** Open‐ended questionnaire items

For the questions below, please think about what information you received about breastfeeding, either directly or through your partner. This could include information from health professionals, leaflets, antenatal teachers, etc. Please think both about what information you received and how it made you feel. 1. What were your thoughts about breastfeeding before your baby was born? And now? 2. What has been your experience of breastfeeding and supporting your partner? 3. Did you receive any information about breastfeeding during pregnancy? What and from where? Was this information useful? 4. What information about breastfeeding would you like to have received during pregnancy? 5. Did you receive any information about breastfeeding after your baby was born? What and from where? Was this information useful? 6. What information about breastfeeding your baby would you have liked to receive after your baby was born? 7. How did information about breastfeeding and breastfeeding promotion make you feel? 8. Can you think of any positive examples of breastfeeding education and promotion you experienced? Why were they positive? 9. Can you think of any negative examples of breastfeeding education and promotion you have experienced? Why were they negative? 10. Do you have any ideas for how breastfeeding should be promoted? What messages would you like to see emphasised and how?

The questionnaire was completed either via a paper copy distributed through local nurseries and mother and baby groups or via an online survey link whereby data was collected through an online questionnaire hosted by survey monkey. Participants recruited through the local groups were also given an online link to complete the questionnaire if required and likewise online participants could request a paper copy. Both the paper and online version of the questionnaire contained and information and consent section and debrief information. Details of how to contact the researcher for further information were included.

### Data collection

For the face‐to‐face groups, permission was initially sought from the group leader or nursery manager. Questionnaires and information were predominantly distributed through mothers attending the group who distributed the questionnaire to their partner. The group leader/manager distributed the questionnaire to mothers who returned it to the group/nursery in a sealed envelope.

For the online version of the questionnaire, permission was sought from the host of various online parenting groups (e.g. http://www.mumsnet.com; http://www.bounty.com). Details of the questionnaire were then posted online with a link to the online version of the questionnaire. Again, study adverts often targeted mothers who passed the information on to their partner as many online parenting sites are frequented by female members. However, adverts were placed strategically to be visible to male audiences (e.g. the parenting site Mumsnet has a board specifically for fathers and attracts male members to the site). To further overcome the lack of direct access to fathers, social media methods such as Facebook and Twitter were used to advertise the study. This enabled the study to be accessed by male participants directly. The benefits and limitations of this approach are considered in the discussion.

## Data analysis

A simple qualitative descriptive approach (Sandelowski 2010) was used. A content analysis was performed for each script. This entailed reading through each script to identify emerging themes. Themes were grouped into key themes and subcategories. All scripts were analysed and coded with a table of key and sub‐themes produced. A random sample of scripts were selected for confirmation of themes by two independent coders.

Proportion of participants who agreed with each key and sub‐theme was recorded in a table to allow an additional descriptive quantitative analysis of data (see Table 2). Data saturation principles were reached for the key themes within 15 participants (Guest *et al*. 2006) and overall the sample size clearly exceeded recommended minimums for qualitative data (Bernard 1995; Creswell 1998). However, smaller sub‐themes continued to emerge throughout the scripts, particularly for negative attitudes or beliefs. The decision to conduct a quantitative count of themes was felt to be a positive approach for this data analysis as this allowed the illustration of such beliefs within the wider sample.

**Table 2:** Themes, sub‐themes and frequency of agreement

Theme	Sub‐theme	*N*	%
Attitude towards breastfeeding	Beneficial to health	111	94.9
	Financial benefits	77	65.8
	Lower responsibility for father	38	32.4
	Little difference between breast and formula	11	9.4
	Concerns about milk supply/sufficiency	42	35.8
	Concerned difficult for partner	31	26.4
	Excluding	68	58.1
Experience of breastfeeding	Health	98	83.7
	Cost	69	58.9
	Convenience and ease	78	66.6
	Excluded from feeding	74	63.2
	Excluded by health professionals	22	18.8
	Conflict	15	12.8
	Helpless/incompetent	72	61.5
	Embarrassed	27	23.0
Experience of education and promotion	Positive example given	65	55.5
	Negative example given	108	92.3
Positive examples	Specific information	54	46.1
	Problem solving	33	28.2
	Inclusion	18	15.4
	Recognition of feelings	16	13.7
Negative examples	Excluded/information only from partner	73	62.4
	Purely on benefits	75	64.1
	Unclear/vague	73	62.4
	Lack of practical advice	81	69.2
	Overstated/guilt inducing	33	28.2
	Patronising	34	29.1
Future support	Direct information to fathers	89	76.1
	Specific evidence‐based information on benefits	67	57.2
	Practical advice on how to support	87	74.3
	Non‐patronising	32	27.4
	Include fathers in promotional material	28	23.9
	Experiences of other fathers	44	37.6

## Results

One hundred and sixty‐two responses were received of which 45 were excluded. Reasons for exclusion included a child outside the age range (*n* = 3) or a partner who did not initiate breastfeeding at birth (*n* = 9). The remaining participants were excluded for partial completion of the questionnaire or responses that were predominantly short answers, e.g. a few words rather than extended responses. The decision was made to exclude for partial completion if participants appeared to have stopped completing the questionnaire (e.g. completed first five questions and then no more) or did not respond to 60% or more of the questions. Responses were considered ‘short’ if repeatedly only a few words in one sentence were used for each answer. This strategy is considered in the discussion.

### Questionnaire completion

Of the remaining 117 participants, responses to the open‐ended items were typically detailed (e.g. more than a few sentences or one or more paragraphs) although length often differed between question items. Responses to the first two questions enquiring about attitudes and wider experiences of breastfeeding often received the longest responses, including reporting of attitudes, emotions and feelings with further questions (e.g. those which asked about specific experiences) often being shorter or more descriptive and factual. Frequently participants would write in detail for the first questions and then refer to those in response to others as they had already voluntarily provided the information before being asked. Responses were typically shorter or absent for the questions asking about specific examples of positive breastfeeding promotion (e.g. participants would state ‘not applicable’ or ‘can't think of any’). However, this was felt to be indicative of general experience, in line with other emerging themes and detail written in other responses. Again, this strategy as a recruitment method is considered in the discussion.

### Sample

Mean age of the 117 participants who were included was 35.63 (SD: 5.12) with a mean years in education of 12.90 (SD: 3.42). Mean age of infant was 39.3 weeks (SD: 24.52). Estimated mean duration of their partners' breastfeeding was 8.45 weeks (SD: 16.10) with a range from 1 to 204 weeks (see Table 3).

**Table 3:** Sample distribution by demographic factors

Indicator	Group	*N*	%
Parity	First‐time father	78	66.6
	Multiple father	39	33.3
Age in years	≤19	3	2.5
	20–24	14	11.9
	25–29	22	18.8
	30–34	45	38.5
	35≥	33	27.5
Education	School	5	4.2
	College	18	15.0
	Higher	67	57.3
	Postgraduate	27	23.1
Marital status	Married	79	67.5
	Cohabiting	33	27.5
	Partner	3	2.5
	Single	2	1.7
Maternal occupation	Professional/managerial	64	54.7
	Skilled	35	29.9
	Unskilled	15	12.5
	Unemployed	3	2.5
Partner any breastfeeding	At birth (*n* = 117)	117	100
	At 6 weeks (*n* = 102)	72	70.5
	At 6 months (*n* = 78)	45	57.6

The majority of participants completed the survey online (*n* = 102; 87.1%). No significant difference was found in demographic background between fathers who completed a paper copy or online.

## Themes

Participant responses were split into four key areas: attitudes towards breastfeeding, experience of breastfeeding, experience of breastfeeding education, information and promotion and ideas for future breastfeeding promotion and education. Within each of these key areas, a number of further sub‐themes emerged. Details of the frequency of men who reported each theme and sub‐theme can be found in Table 2.

### Attitude towards breastfeeding

Men revealed how they felt about breastfeeding both before their baby was born and now. The majority of the men felt positive, with all men reflecting at least one benefit of breastfeeding. Men were encouraging that their partners tried to breastfeed their baby when it was born, although ultimately referred to it as her choice. Rationale for this mainly centred on the health benefits of breastfeeding, a factor raised by nearly 95% of men within the questionnaire.

I really hoped ***** (name of partner) would breastfeed as I'd heard so much good stuff about it. Breast is best.

I hadn't thought about it much until she was pregnant but then reading about it I was very positive.

However, men also made reference to wider benefits of why they were supportive of breastfeeding. Lower cost and lower responsibility especially in terms of night feeds were made. Many of these were made with a humorous tone evident by the use of exclamation marks or smiley faces added after their response

Breastmilk is free … both my wallet and I wanted her to choose it!

Let's see … getting up in the night versus making supportive noises and going back to sleep? No competition!!

However, a small proportion of the men (9%) were more ambivalent in their thoughts about how their baby should be fed. Many did not really see a difference between the choices and others felt that it was not their choice to make. Indeed, a common theme throughout the responses was that men did not feel they could encourage their partner to breastfeed; it was her choice.

I didn't see much difference between formula and breast milk. My friends had bottle‐ fed and they seemed fine. But she really wanted to and I'm glad she did.

I didn't feel strongly either way. However my wife wanted to breastfeed and everyone else told her to so she did … I didn't see how I could tell her what to do.

Although the majority of men referred to breastfeeding in a supportive manner, men did also hold concerns about the choice to breastfeed. Reasons behind these thoughts appeared quite similar to the literature exploring why women choose not to breastfeed. Fathers held concerns that that breast milk would not be good enough or that it would be very difficult for their partner. This was often based on experiences heard from other fathers.

I worried that it wouldn't be enough for him or something would be wrong. Formula seemed logical – nothing wrong with it.

I'd heard from friends that their wives struggled and it hurt and was really difficult. I didn't want her to do that.

Others however were concerned that they would be excluded from the feeding relationship. Fathers saw feeding as a positive part of bonding with their baby and wanted to get involved.

I wanted to feed him so thought we would bottle feed. I was sad at the thought that I couldn't join in.

### Experience of breastfeeding

Moving on, fathers described their experience of their baby being breastfed. Unanimously, fathers reported both positive and negative experiences. The main themes within their responses focused on the health benefits of breastfeeding, alongside issues of low cost, convenience and ease.

#### Health

Fathers were very strong believers that their baby had benefitted from being breastfed. They felt that their baby was ill less frequently and was generally healthier and more content because of breastfeeding.

She thrived on it. Put on weight, looked happy, rarely ill.

I'm sure she gets less ill than other babies. She's in nursery and the other parents always say how often they have to pick them up because they're ill. She's rarely ill.

#### Cost

Cost also featured very strongly in fathers positive reflections on breastfeeding. Fathers referred to both personal costs and wider savings from prevention of illness.

Breastfeeding was great. It's free for a start!

I did like the fact I didn't need to fork out for formula.

#### Convenience and ease

A number of fathers raised the idea that although difficult at first, breastfeeding once established felt like an easy and convenient option. Fathers didn't have to worry about preparing bottles or running out of milk.

I have mates who bottle fed their babies and they had to worry about taking bottles out, heating them up. We just left the house.

It was hard at first but then it seemed really straightforward and easy. Thought I have to admit it wasn't me feeding her was it!

However, all of the fathers to some extent reflected on the negative experiences of breastfeeding. Although they emphasised how positive they felt the breastfeeding relationship was, there are many aspects of their experience that could be addressed.

#### Excluded

One of the central themes included how the fathers felt in relation to their own experiences at this time. Fathers reported feeling excluded and dismissed and as if they were not part of this special time. Although many realised it was not intentional, they felt that breastfeeding encouraged the mother to focus on her baby leading to them feeling isolated from this time and relationship.

I was jealous at some points. He seemed to enjoy feeding. He either slept or cried the rest of the time but feeding was the happy bit. I couldn't do that.

Feeding takes up a lot of time when they are young. I joked my partner was permanently attached to another man. I was half joking but I did feel a bit put out which sounds terrible now. She wasn't too impressed either!

This included feeling dismissed by health professionals as if they were not an important part of the decision or experience.

I asked the midwife what I could do to help my wife. She said cook her dinner, bath the baby and so on. I understood that but I wanted to help and join in with the feeding experience and I couldn't. I was annoyed.

When I said to a nurse I felt helpless she said enjoy the break and laughed like it was nothing to do with me.

#### Conflict

A number of men did admit to feeling negative emotions around the breastfeeding experience that led to conflict with their partners. Although they admitted that this could be to do with the wider newborn experience, men reported feeling frustrated and angry at being excluded or feeling that their opinion did not count.

I'm really ashamed at it now but I did take it out on my partner sometimes by being miserable with her or even shouting sometimes. I felt excluded and stressed but it wasn't her fault. She was only doing what was best for the baby.

It did cause some arguments which I know wasn't fair. But I felt useless and like she didn't want me any more. I didn't see why she couldn't give him a bottle.

#### Helpless

However, related to this, fathers also described negative emotions in relation to supporting their partner and child. Men reported feeling anxious, helpless and guilty that they were unable to help their partner with feeding or overcome their problems for them.

My wife was in tears and in pain. She was exhausted and I didn't know what to do.

It is really hard to see someone you love struggling and not to be able to do anything.

Connected to this a number of fathers also raised the idea that they felt incompetent in caring for their baby because they could not feed them. Breastfed babies often refused a bottle or only wanted to be comforted by the mother because of that special feeding relationship.

He kept crying. All he wanted was feeding. Not much I could do which made me feel rubbish.

My partner was fed up and wanted to go out for a bit with out the baby but it was impossible. He wouldn't take a bottle. He wanted to feed every hour or so sometimes. I wanted to help but couldn't.

#### Embarrassed

Although this factor was less common in the responses than many, fathers did also raise the idea that they did feel embarrassed and self‐conscious to watch their partner breastfeeding their baby in front of family members or in public. However, the majority of fathers who reported this feeling also described how this feeling passed with time and became less of an issue.

At first I freaked out about her feeding in front of people. I couldn't stop thinking that she had her breast out in front of my father or my friends and that they were getting an eyeful. Thankfully I grew up though and realized you couldn't really see anything and it was better than the screaming!

### Experience of information and advice

In relation to their experience of their baby being breastfed, fathers were asked about their experiences of information and advice about breastfeeding before the birth. This included indirect information that was passed on to them through their partner and any information that was specifically directed towards them. Overall, few fathers reported getting detailed information directly from a health professional or antenatal teacher. Most described information being passed through their partner in the form of leaflets or conversely little information reaching them at all.

I don't recall breastfeeding ever being discussed in front of me.

There was a leaflet that the midwife gave my wife. There were some posters up too in the doctors.

Many reported how their opinion of breastfeeding was based on information their partner repeated to them, although a number of fathers did choose to do their own research and reading on the subject.

My wife told me about it and she wanted to do it so I supported that really.

I went on the internet and did some reading myself.

Fathers were also asked about their reaction to the information they did receive from health professionals and others sources such as leaflets, posters and antenatal teachers. A minority of fathers did describe positive examples of information and advice they had received. Mainly, this centred on specific reasons why breastfeeding is good rather than general advice and again specific ways in which they could support their wives.

#### Specific information

A number of fathers noted that they liked very specific information when it came to breastfeeding. This helped them believe in the promotional literature as it was supported by evidence.

I read somewhere that if you breastfed you saved £500 a year on formula and bottles and things and were saving the NHS money too. I like figures.

Our antenatal teacher gave us a leaflet saying that if you breastfed for two days then you help prevent some illness and then if you did it for a week you prevented something else. It made a good reference point when encouraging my wife to continue.

Conversely, fathers disliked information that was unclear, vague or did not give them practical advice on how to care for their partner

The leaflets my wife got given all said breast is best but they didn't say why. Some made vague reference to tummy bugs but it was all a bit basic. I wanted to know why so I knew why it was worth it!

I wanted information on how to help my partner. There was nothing on that.

Linked to the provision of detailed information was the belief that information regarding breastfeeding should not be overstated. Although men recognised that breastfeeding had many health benefits they felt that many messages placed too much pressure on new mothers, often with a lack of practical support or guidance once the baby was born. Men felt helpless when their partner felt upset or guilty about wanting to stop breastfeeding and were annoyed at the level of guilt placed on new mothers.

My partner got very distressed when she gave up breastfeeding that she was harming our baby. I could see he was fine but she felt really guilty. It wasn't fair.

I got quite angry at people telling my wife she had to breastfeed when they couldn't give me evidence that it wasn't as catastrophic to formula feed as they implied.

#### Problem solving

A positive experience that was frequently recalled was when men received practical information on how they could support their partner. This made them feel confident and positive about breastfeeding.

We had a great antenatal teacher who did troubleshooting with us. How to do it … how to solve this problem or that problem. I felt I knew what to do and could do something when things went wrong.

The midwife suggested how I could sit with my wife and baby during feeding. It made me feel closer and then happier to support it.

Fathers who gave negative examples tended to discuss how information focused purely on the benefits of breastfeeding rather than enabling them to be supportive.

Most of the information seemed to focus on why breastfeeding was a good thing. There wasn't much on how to do it.

I knew it was best but I wanted to know how to support my wife to do it. That information wasn't there.

#### Inclusion

Predominantly, men wanted to be included in breastfeeding education and enjoyed taking part in information sessions. They benefitted from discussing breastfeeding with both professionals and other fathers in the same situation.

We went to both NHS and private antenatal classes. The private one was good as she treated us like parents not separately as mother and father. Dads were important and included too.

Our antenatal class included dads in the breastfeeding discussion. I heard from other dads this was rare but it was good to feel part of it and get that information.

Conversely, a high proportion of fathers reported how they felt that they had been subtly or more directly excluded from breastfeeding information sessions or felt that they had not been considered within the experience.

The information was all aimed at my wife. What she could eat, do, experience etc. I know she was the key player here but I felt that it was nothing to do with me.

When we went to antenatal classes they did a session on breastfeeding. They sent all the dads down the pub that night.

#### Recognition of feelings

Linked to feeling included in information and advice was admiration for professionals who also recognised the fathers' feelings at this time. Fathers wanted to feel that their emotions and feelings were being recognised.

One midwife turned to me and asked how I felt about feeding. It was great to be acknowledged in all of this.

However, a number of fathers reported that they felt patronised by the way they were addressed by professionals. The suggestion was that they needed to be bribed or convinced that breastfeeding was a positive thing through seeing benefits to themselves.

There was this poster up in the ward apparently trying to promote breastfeeding which told dads it was a good thing because they would get more sleep. It was awful – not all dads are lazy and uninvolved which is what I felt it was implying. I wanted to help.

One midwife actually told me in front of my wife that breastfeeding was a good thing as it would make her breasts bigger. I'm not that shallow.

Overall, fathers appreciated information and education that included them, was specific and detailed and provided them with practical ways in which they could help their partner. Unclear, patronising or excluding information was seen in a negative light. From this, fathers suggested ways in which they would like to see future breastfeeding education and promotion shaped to promote paternal involvement and equip them with sufficient knowledge and understanding in this area.

### Future support

Overarchingly, fathers were positive that men should be included in breastfeeding promotion and advice, as even if they were not directly responsible for feeding, or even decision making when it came to breastfeeding, they needed to be armed with facts and guidance of how to support their partner. The concept of not excluding new fathers from any part of their infants' lives was also raised.

Dads do need information. Just so they know why and how to support their partner.

It's a big part of early parenthood. It's important not to exclude dads.

Fathers detailed a specific number of ways to include new fathers in breastfeeding education. Predominantly, ideas were split into two main themes – what information they wanted and how they wanted to receive it. A number of suggestions directly reflected their positive and negative experiences above. Men wanted evidence‐based information that not only promoted breastfeeding but gave them guidance of how to support their partners when problems arose.

I don't want to know that it is best. I want to know why and specifically why so I can feel we're making the right decision.

I'd want to know how to support my wife when she found it tough. When to know there is a problem and what to do. Not just why you should breastfeed.

Fathers also wanted to be included in the information delivered to them as if they were an important and competent decision maker.

I would like be included in decisions that affect my son. Just because I can't feed him doesn't mean I'm not interested.

I don't need to be bribed with stupid suggestions that it will mean I don't have to get up in the night. Give me real facts.

However, in addition to this, fathers also gave some specific ideas as to how they would like to see future breastfeeding promotion and education targeted and made appropriate and useful for new fathers.

#### Give information about what breastfeeding is really like

Alongside practical guidance, fathers called for clearer information about what they could really expect from breastfeeding. They wanted to be forewarned about potential issues and that breastfeeding might not be simple. Many first‐time fathers recalled feeling shocked that it was not the simple experience they felt they had been led to believe.

I thought it was going to be simple. They told us it was best for the baby so I didn't expect problems and then felt helpless.

One thing I didn't realize at the time was that the constant feeding was only a short bit of time. It passed. We've kind of forgotten about it but it didn't feel like that at the time. If I'd known or thought about it I would probably have been more supportive.

#### Include fathers in promotional material

Specifically, a number of fathers noted that they would like to see more father‐friendly promotional material in terms of the inclusion of fathers within it. This included both literature aimed solely at fathers (or a section within material) to recognise their importance or additionally through more subtle ways of including pictures of fathers or whole families within such literature.

I would like to see photos of families used or dads in breastfeeding literature.

Perhaps a leaflet about breastfeeding aimed at fathers – how they can support their partner and so on.

#### Experiences of other fathers

One way in which this could be delivered was the idea of giving more experiences of other fathers. Dads wanted to hear about how other fathers had felt and what they had done to support their partners at this time, for example, how they coped with different problems or how they overcame feeling embarrassed at their partner feeding in public.

Not many of my friends had babies and they tended to bottle‐feed. I wanted to know how other dads felt and whether they felt excluded or fed up.

I'd have liked to know that other fathers felt this way too.

Overall, fathers within this sample felt very strongly that they wanted to be included in breastfeeding promotion and education. They wanted factual advice and information about how to solve different problems delivered in a relevant and non‐patronising format.

## Discussion and conclusions

This paper explores fathers' experiences of supporting their partner to breastfeed and their attitudes towards breastfeeding education and promotion. Consistent with previous research in the area, the findings show that fathers are willing and enthusiastic about supporting their partners to breastfeed. However, men reported feeling excluded and helpless at this time, wanting more specific information, inclusion and guidance on how to support their partners. Fathers wanted more father centric breastfeeding education and wished to be recognised as important and competent advocates and enablers of their partners' decision.

Paternal inclusion in educational breastfeeding programs has been shown to result in mixed outcomes in relation to increasing duration and breastfeeding rates (Lovera *et al*. 2010; Susin & Giugliani 2008; Maycock *et al*. 2013). Nevertheless, a developing body of evidence clearly identifies that the support of fathers does play an important role in mothers feeling supported to breastfeed (Gage & Kirk 2002; Ingram *et al*. 2002; Garfield & Isacco 2006), and these findings have implications for health professionals and those working in health promotion. Fathers want to be included in breastfeeding education and want to be taught to identify and solve problems for their partner, all of which may be an important step in increasing breastfeeding rates in the UK and elsewhere. Ensuring this may be perceived as being in keeping the wider global trend of involved fatherhood (Lamb 2000; Flouri 2005) and a worldwide policy that seeks to highlight and take account of the contribution fathers make to their baby's care and health outcomes (UN 2011). The findings are thus important in considering how breastfeeding education and support for fathers can move forward, and be incorporated into future strategy.

In line with previous research, the findings showed that fathers were interested in breastfeeding and wanted to be involved in the experience (Sherriff *et al*. 2009; Tohotoa *et al*. 2009; Sherriff & Hall 2011). They were supportive of breastfeeding particularly for reasons of health and convenience, reflecting findings by Mitchell‐Box & Braun 2012). However, their experience of supporting their partner at this time was not always positive. Although they recognised the importance of breastfeeding, they often felt excluded, embarrassed, anxious or unsure of how to help, which appear to be common themes in the literature (de Montigny & Lacharite 2004; Pontes *et al*. 2009; Tohotoa *et al*. 2009; Henderson *et al*. 2011; Mitchell‐Box & Braun 2012). These findings are of concern as paternal involvement and support for breastfeeding has repeatedly been show to increase breastfeeding duration and initiation (Scott *et al*. 2001; Gage & Kirk 2002; Garfield & Isacco 2006), likely through increasing maternal self‐efficacy and confidence (Hauck 2004; Swanson & Power 2005; Hauck *et al*. 2007; Mannion *et al*. 2013).

Specifically, fathers were keen to be given education and information about breastfeeding in relation to recognising and overcoming different breastfeeding problems reflecting previous research (Sherriff *et al*. 2009; Sherriff & Hall 2011). Men frequently report feeling helpless during pregnancy, childbirth and the newborn period as they cannot solve their partners' problems (Bäckström & Hertfelt Wahn 2011; Steen *et al*. 2012; Miller 2013) and want to be able to provide practical support to their partner (Datta *et al*. 2012). In the current sample, men reported feeling helpless as their partner faced breastfeeding difficulties or exhaustion and were unsure how to help or support her. They suggested that breastfeeding education should be expanded to include fathers, particularly equipping them with the skills they need to support their partner through difficulties. Indeed, previous findings suggest that men view their role in the breastfeeding experience as being there to problem solve and find solutions (Rempel & Rempel 2011). Initial research has shown benefits to educating fathers during the antenatal period. One small trial taught father to identify and solve breastfeeding problems and reported an increase in breastfeeding rates at 6 months (Piscane *et al*. 2005). Another trial offering fathers a 2‐h breastfeeding antenatal class and post‐natal support found that breastfeeding levels were increased at 6 weeks post‐partum (Maycock *et al*. 2013). Finally, another small trial inviting fathers to attend breastfeeding antenatal classes alongside their partner increased the duration of exclusive, but not overall, breastfeeding (Susin & Giugliani 2008).

Fathers also reported wanting more specific and accessible information about the benefits of breastfeeding. This echoes previous work suggesting that specific details and facts are important to fathers in lieu of more general and basic information (Sherriff & Hall 2011). Perhaps, literature or education could incorporate specific evidence‐based intervention. Data calculating the number of infants who could be prevented from being ill (Ip *et al*. 2007) or the economic savings brought about by breastfeeding (Renfrew *et al*. 2012) is easily available and would fit well with the fathers ideas.

Men in the sample also wished to be recognised by professionals and literature as an important part of the breastfeeding decision and relationship. Although, as in previous research, fathers believed that the decision to breastfeed ultimately lay with their partner (Avery & Magnus 2011; Datta *et al*. 2012; Sherriff *et al*. 2013), they wanted their role and emotions both specifically as a supporter and more generally as a parent to the infant to be recognised and supported. This included being involved in antenatal education such as breastfeeding classes through to visual representation in the literature, as has previously been suggested by fathers (Sherriff *et al*. 2009, 2013). Listening to the paternal voice here is important. If we want fathers to be involved in supporting mothers to breastfeed, greater resources need to be channeled to supporting them both in terms of information and recognising their thoughts and emotions at this time. There is little cost involved in opening breastfeeding education up to men and thinking about the fathers needs and viewpoints when discussing breastfeeding, with countries such as Australia actively involving men in the process (Schmied *et al*. 2002). However, delivery of this support needs consideration. Women may feel embarrassed at receiving breastfeeding education in a class with men because of societal connotations of the breast as sexual (Henderson *et al*. 2011) or there may be cultural implications in their inclusion (Molzan Turan *et al*. 2001). Additionally, men and women can hear information in different ways or have different preferences for information delivery or content (Buist *et al*. 2003). Separate antenatal classes aimed at fathers have been viewed as beneficial (White 2007). Involving and supporting fathers in breastfeeding by health professional is also part of the broader and international policy trend of ‘involved fatherhood’, which seeks to involve fathers in all aspects of their baby's/child's health and well‐being.

Caution should however be given to the level of paternal involvement. Although paternal involvement in the parenting process has been shown to have numerous benefits for the child (Lamb 2010), it is possible that too much control and involvement may have a negative impact on the mother – infant bond and thus breastfeeding duration. One study examining level of paternal involvement and breastfeeding duration found that where fathers are highly involved in the early care of their infant, breastfeeding levels were lower (Ito *et al*. 2013). Potentially, fathers wanting increased involvement may lead to feeding cues being missed, separation of mother and infant or increased bottle use all of which may lead to reduced milk supply and breastfeeding cessation (Dewey & Lonnerdal 1986; Daley & Hartmann 1995; Thulier & Mercer 2009). However, the correlational nature of the study should be noted – it is possible that if a mother chooses to formula feed her partner then becomes more involved in infant care with this possibly being one of the driving forces of feeding choice (Brown *et al*. 2011a). In the current study, fathers consistently raised the issue of feeling excluded from the relationship. Emphasis should be placed on finding a balance by encouraging fathers to participate in wider parental care or to be a supporter during feeds, rather than necessarily giving a bottle of formula. Research suggests that fathers enjoy supporting the mother, and caring for the infant, in other ways (Sherriff *et al*. 2013), thus perhaps explicit ideas on how to do this could be discussed with fathers antenatally.

The research does have its limitations. Firstly, the sample was self‐selecting, potentially leading to only the most motivated fathers taking part. Likewise, mothers who were particularly motivated to take part either through positive attitudes towards breastfeeding or through negative experiences may have been more likely to encourage their partners to take part. Fathers were also older and had a higher level of education than average. Breastfeeding duration of their partner was longer than average (DH 2013) but this would be excepted in a sample where breastfeeding was initiated by all, and a clear interest found. Care should be taken in considering whether the findings are generalisable to a wider population. Further research should explore the issue in a wider population based sample.

Secondly, methods of recruitment were non‐traditional because of fathers being a hard‐to‐reach group. Research directly with fathers is sparse; mothers are often recruited to studies exploring parental behaviours and attitudes, often because they are perceived to be the parent most involved in the child's care (Phares *et al*. 2005; MacDonald *et al*. 2010; Shapiro & Krysik 2010; Johnson & Simpson 2013). Fathers are also more difficult to access as typically they return to work after the birth, do not have child‐related health appointments and are less likely to attend baby groups and other organised activities. Indeed, much of the research with fathers involves smaller numbers (e.g. Arockiasamy *et al*. 2008; Deave & Johnson 2008; Premberg *et al*. 2008) accessed through health professionals or specific parenting initiatives, which in itself might bring about bias (e.g. because of motivation to attend or reason driving attendance such as being part of an at‐risk group).

Although a minority of participants were approached directly face to face because of their direct participation in parenting groups (*n* = 6), identifying and contacting fathers predominantly had to be non‐traditional. Two main methods were used for this: mothers as gatekeepers and online recruitment. Recruitment using these methods was successful in terms of sample size gained in a efficient format and in a short time frame. However, it does have its limitations. In terms of using mothers as gatekeepers, bias may arise because of the selection being driven by the motivation and attitude of the mother to pass on study information and relationship between mother and father (e.g. pressure to take part, reluctance to disclose information). It is also of course likely to exclude separated fathers.

The second approach used was to utilise online methods, advertising the study on parenting forums both to mothers (again as gatekeepers) and directly to fathers. A variety of parenting boards were used aimed at parents, with specific adverts also places on boards aimed specifically at fathers. This method was successful in yielding a high number of responses although it is unclear whether these responses were prompted through mothers or fathers directly. In recent years, use of Internet forums among pregnant and new mothers has increased (Hall & Irvine 2008; Plantin & Daneback 2009) and they are now typically used by a wide spread of demographic groups (Quan‐Haase *et al*. 2002; Sarkadi & Bremberg 2005). Fathers are also starting to use parenting forums for social support and information seeking including those aimed generically at ‘parents’, just fathers and including ‘trespassing’ into traditional female environments such as Mumsnet (Fletcher & StGeorge 2011; StGeorge & Fletcher 2011; Eriksson & Salzmann‐Erikson 2013).

Internet‐based recruitment is growing in popularity in health research (e.g. Alcalde 2011; Ferguson & Hansen 2012; Hamilton *et al*. 2012) as it allows access to a targeted sample in a cheap and effective way (Koo & Skinner 2005). However, it does have its limitations. Internet users may however be a select, well‐educated and proactive group (Drentea & Moren‐Cross 2005), and indeed, fathers who specifically choose to use parenting forums might be particularly motivated in their fathering role.

Moreover, although using an online questionnaire format may encourage participation as it is anonymous and simple and immediate to complete, it does remove the researcher from the process. Prompts for more information or clarity cannot be given leading to only partial completion of a questionnaire or insufficient detail. This is a clear limitation to the data collection process, as it led to a number of participants being excluded. However, weighed up against the number of participants it did recruit, who may not have been accessible or willing to complete a more lengthy process, it was felt to be a successful method. Future research might like to examine ways to increase completion of such questionnaires in more detail, or to include the option of responding to the participant to ask further questions or gain clarification if this was appropriate. Indeed, further research should generally explore methods of involving a wider sample of fathers more directly in research projects, both from the perspective of a more generalisable sample and to develop new ways in encouraging fathers to actively take part in research.

## Conclusion

The findings shows that fathers in the study expressed a clear interest to be involved with and support their partners through the breastfeeding experience. However, they felt they lack the knowledge, understanding and skill to do this and called for more education and support to be directed towards them, rather than their partner alone. The findings have important implications for those working to support new families, and to consider the future of breastfeeding education, promotion and policy.

## Source of funding

Amy Brown was supported by an ESRC Postdoctoral Fellowship.

## Conflicts of interest

The authors declare that they have no conflicts of interest.

## Contributions

AB was responsible for study design, data collection, data analysis, report writing and critical amendments to paper. RD was responsible for report writing and critical amendments to paper.
